# Supplementary material for: Reconstructing single-cell resolution from spatial transcriptomics with CellRefiner
Source: Nat Commun. 2026 Feb 27;17:3304. doi: 10.1038/s41467-026-70090-2 (PMC13066420; doi:10.1038/s41467-026-70090-2)
Supplement: Supplementary file 1 — Supplementary Information [file 41467_2026_70090_MOESM1_ESM.pdf]

Supplementary Information

Reconstructing single-cell resolution from spatial transcriptomics with CellRefiner

Eric Bourgain-Chang, Xiangyu Kuang, Zixuan Cang, Qing Nie

Supplementary Figures

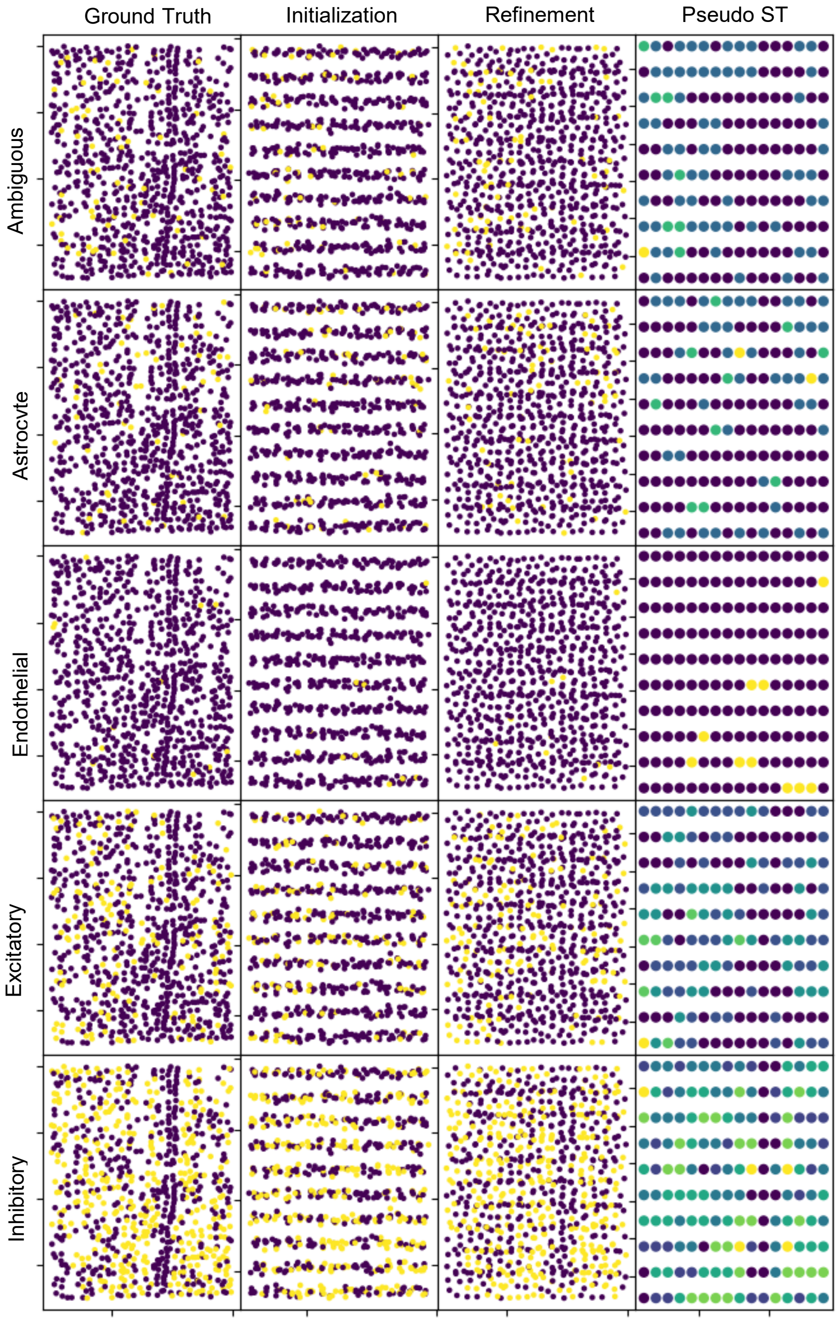

Supplementary Figure 1

MERFISH Extraction

Extracted region from a MERFISH slice from the mouse hypothalamic preoptic region used as ground truth across various cell types. Cells corresponding to each respective cell type are in yellow with the rest in purple. For the pseudo ST spots, intensity corresponds to proportion of corresponding cell type in each spot. Cell types with low number of cells (<20) are excluded. These outputs use the known mapping between MERFISH and pseudoST data constructed from the MERFISH data.

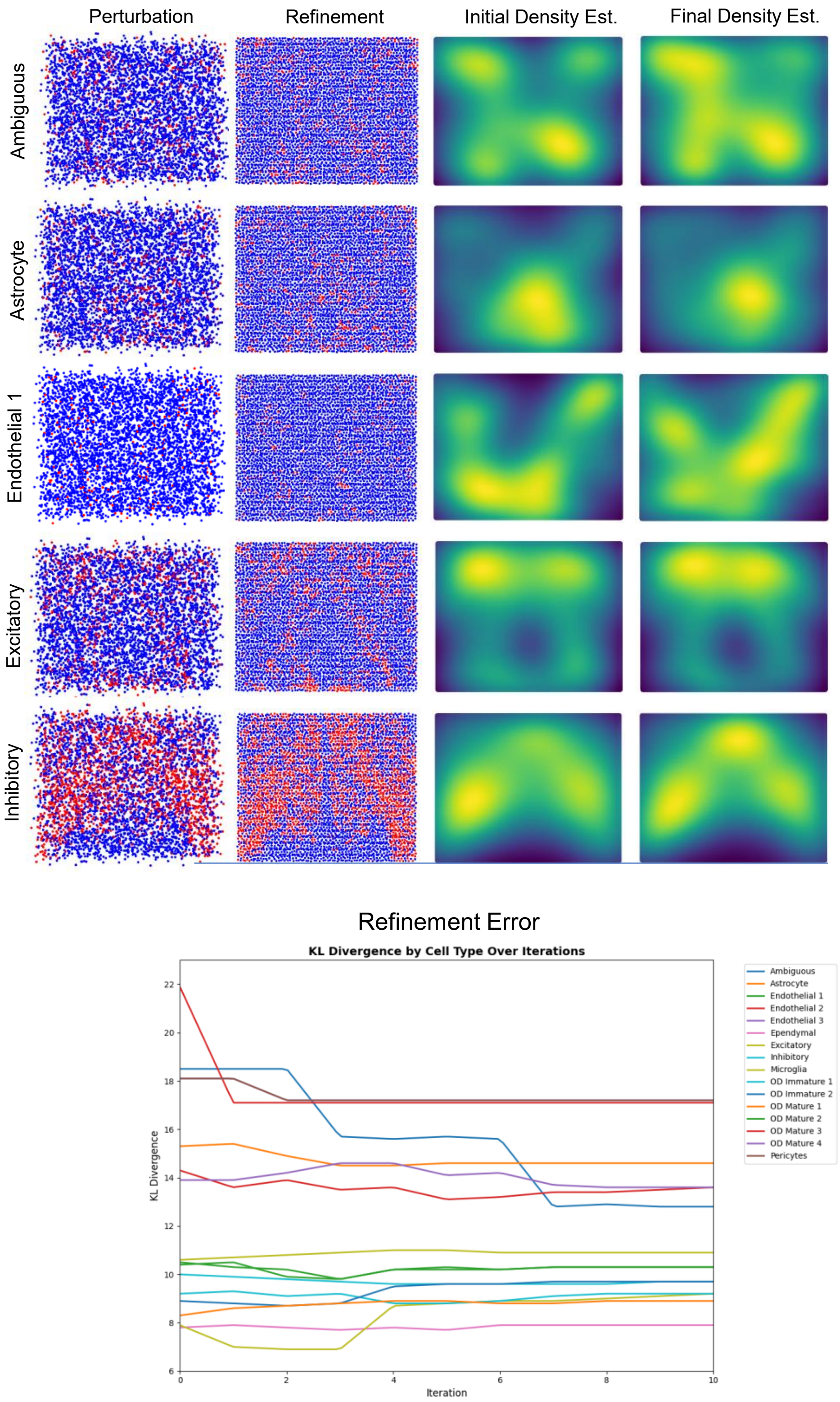

Supplementary Figure 2

MERFISH Testing

Entire MERFISH slice from the mouse hypothalamic preoptic region used as ground truth across various cell types. Cells corresponding to each respective cell type are in red with the rest in blue. Density is calculated using a Gaussian kernel and used to find KL divergence with ground truth over iterations. These outputs use the known mapping between MERFISH and pseudo ST data constructed from the MERFISH.

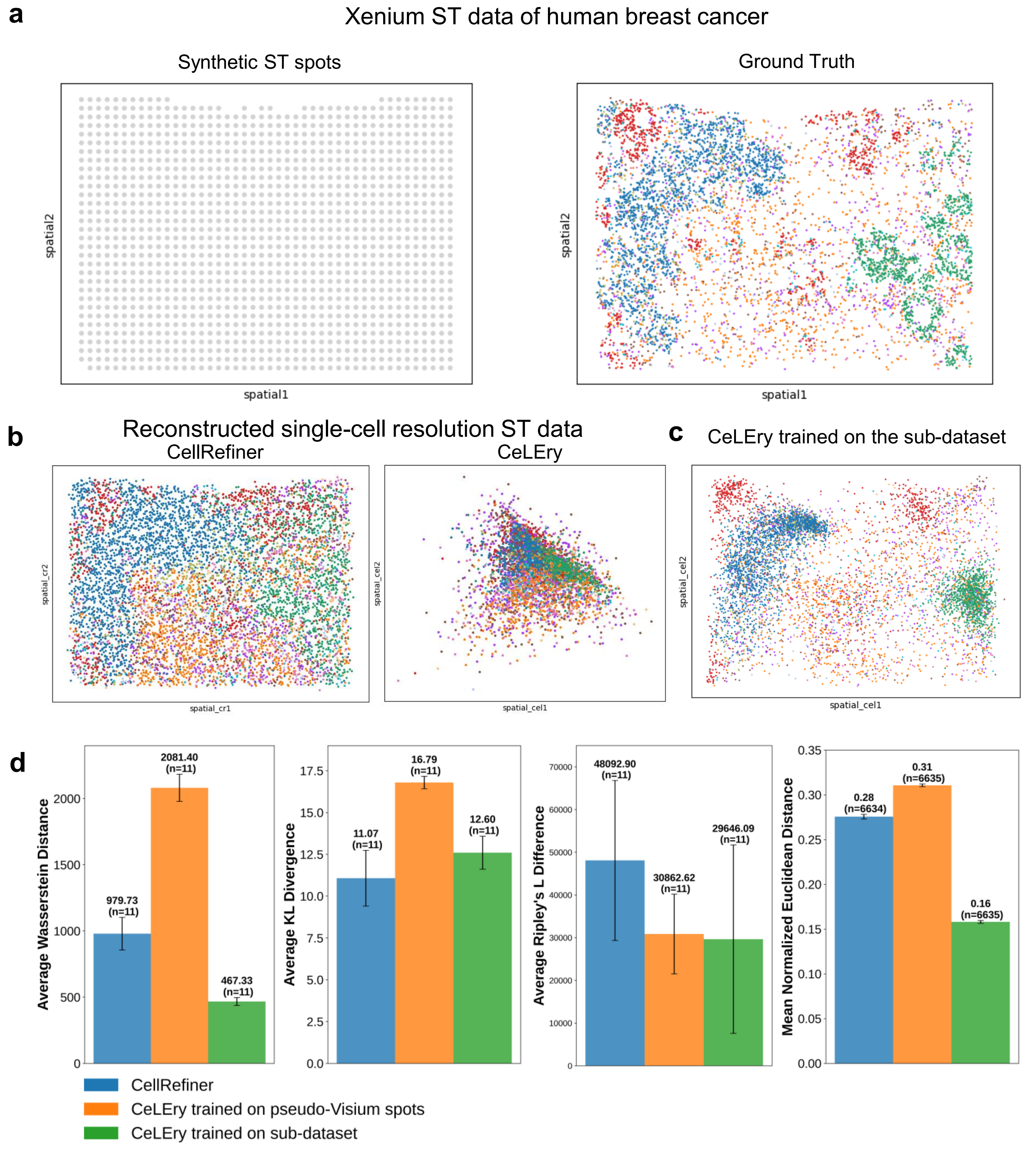

Supplementary Figure 3

Comparison of CellRefiner and CeLEry in reconstructing single-cell spatial distributions using the Xenium human breast cancer dataset

a, Cells from the Xenium ST data aggregated to form pseudo-Visium spot data used as the low-resolution input for the reconstructing single-cell spatial distributions, and the ground-truth cell positions from the Xenium ST data with dot colors represent Leiden clustering. **b**, Single-cell spatial distributions reconstructed by CellRefiner and CeLEry, where CelEry is trained on the pseudo-Visium spot data. **c**, CeLEry-reconstructed single-cell spatial distribution obtained by training the model on the Xenium sub-dataset. **d**, Comparison of reconstruction accuracy using four error metrics between the reconstructed and ground-truth cell positions. The mean values for each metric with sample sizes are presented. Bar plot element: bar height, mean; error bar, standard error of the mean. Source data are provided in the Source Data file.

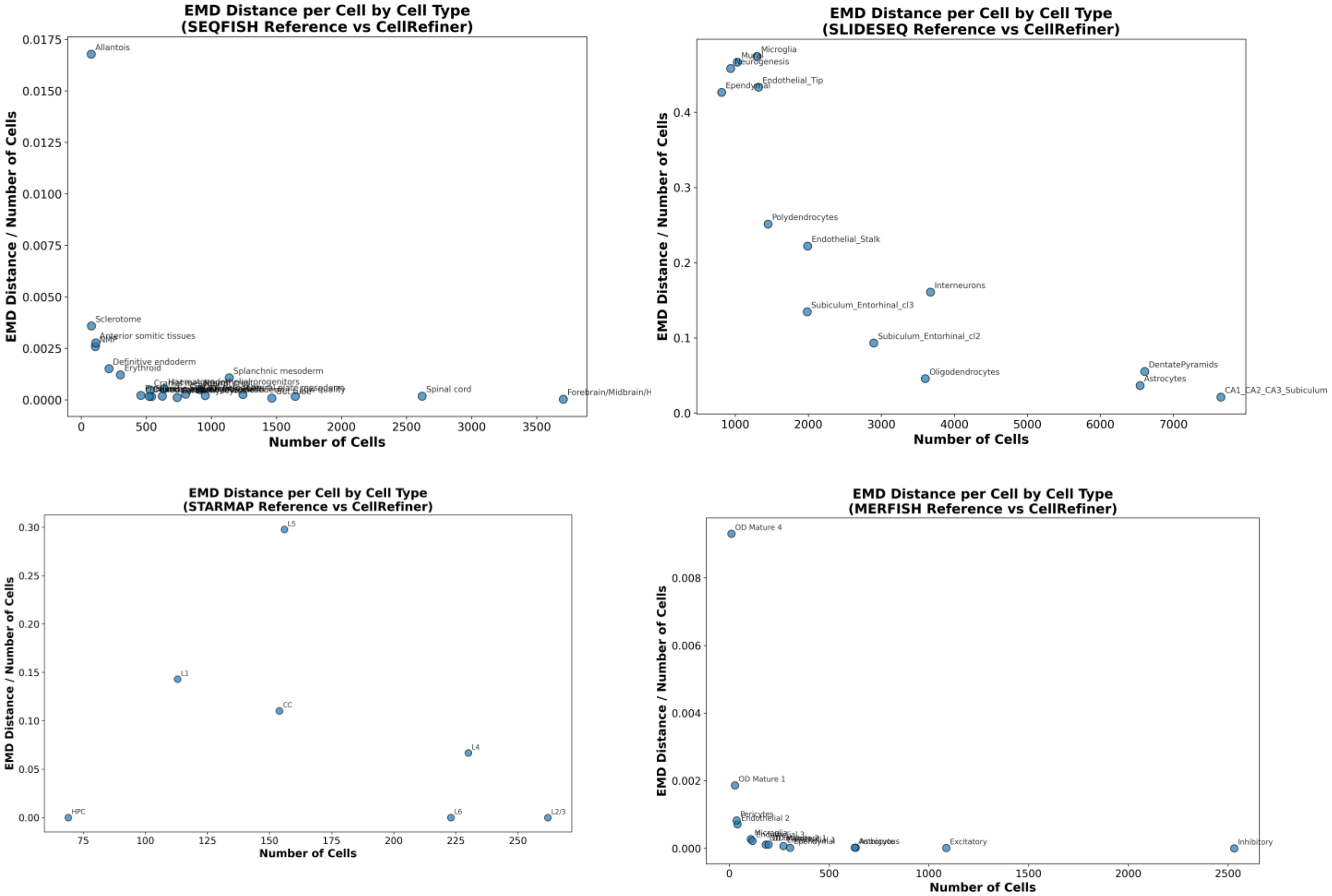

Supplementary Figure 4

Performance of CellRefiner on different cell type sizes.

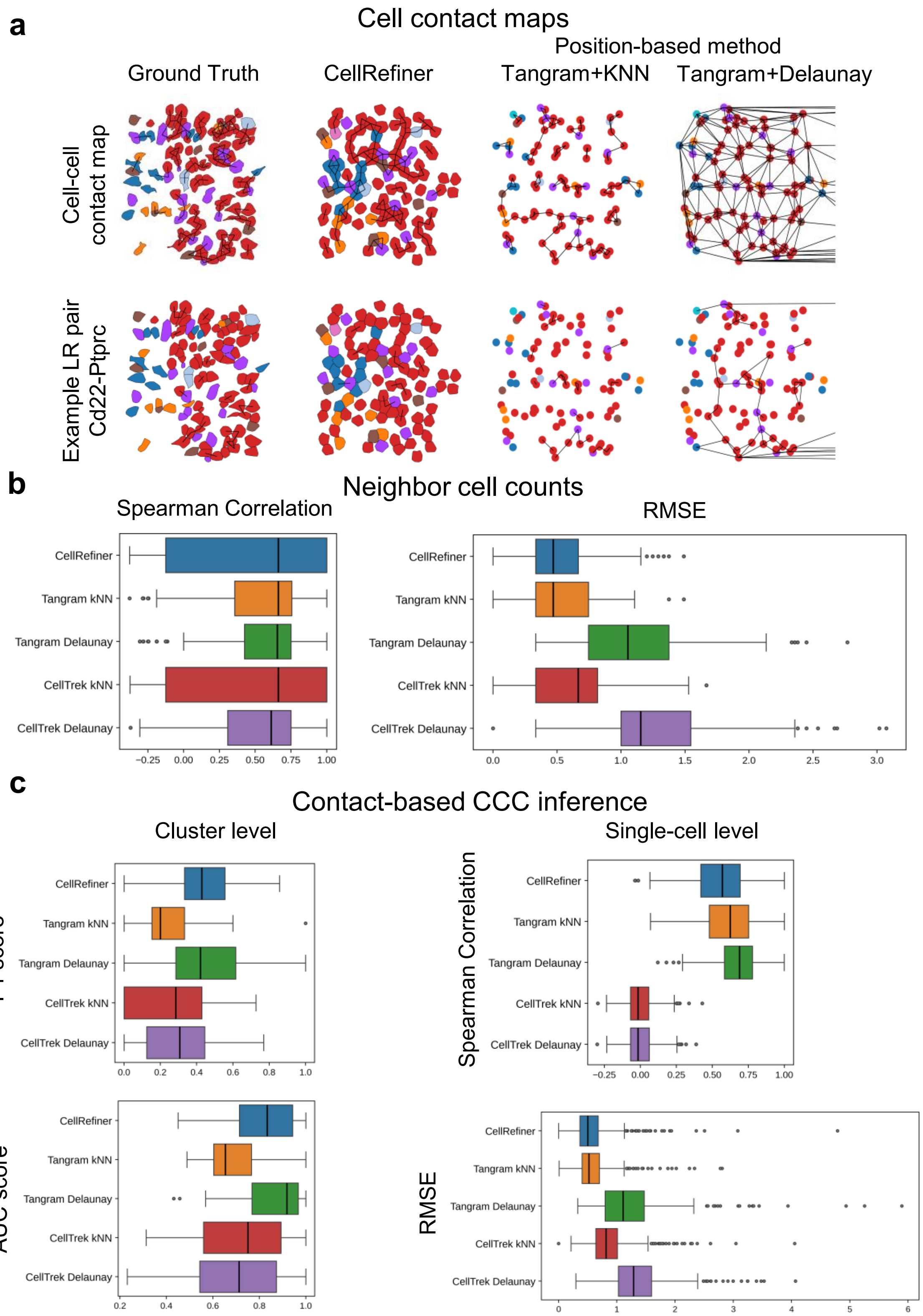

Supplementary Figure 5

Evaluation and comparison of contact-based CCC inference

a The ground-truth cell contact map of original seqFISH+ data and the reconstructed maps by CellRefiner and a representative single-point approach Tangram paired with KNN graphs or Delaunay graphs. **b** Evaluation of predicted neighboring cell type composition of each cell on the cell contact map. **c** Evaluation of predicted contact-based signaling at cell cluster level (evaluated with F1 and AUC scores) and single-cell level (evaluated with Spearman's rank correlation and root mean square error). Boxplot elements: center line, median; box limits, upper and lower quartiles; whiskers, 1.5x interquartile range; points, outliers; sample size, n=508. Source data are provided in the Source Data file.

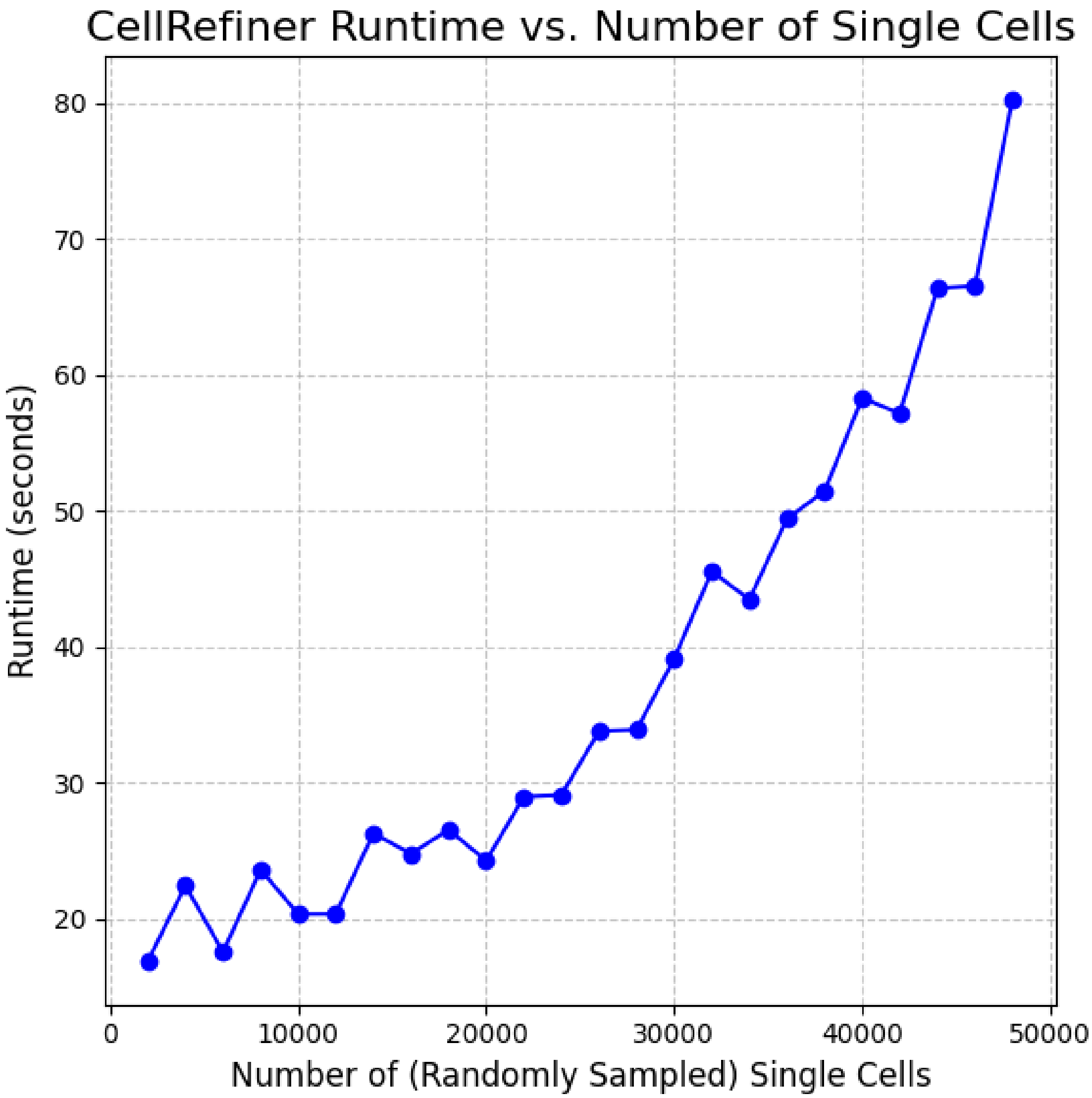

Supplementary Figure 6

Computational cost

The computational run time of CellRefiner against varying number of modeled cells. The numerical experiments are conducted on a computer with AMD Ryzen Threadripper PRO 7985WX 64-Cores Processor and NVIDIA RTX 6000 Ada Generation.

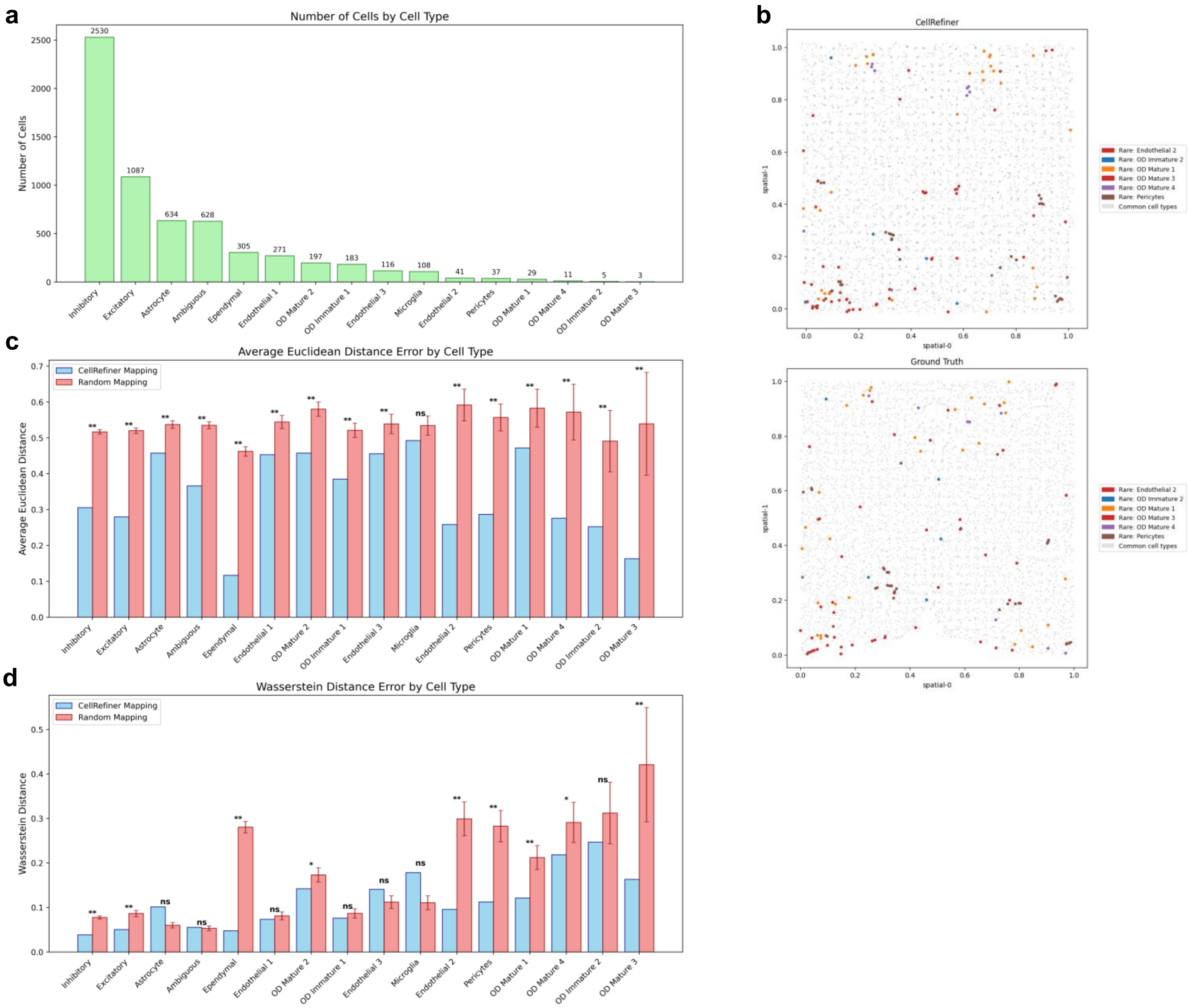

Supplementary Figure 7

Evaluating performance of CellRefiner on rare cell types

a, Distribution of counts for cell types in MERFISH mouse hypothalamic preoptic region. **b**, CellRefiner output for cell types with low counts along with the ground truth. **c**, Euclidean distance error for CellRefiner mapping (blue) versus random mapping (red, n=200). **d**, Wasserstein distance error for CellRefiner mapping (blue) versus random mapping (red, n=200). Bar plot element: bar height, mean; error bar, standard deviation. Significance levels: **p < 0.01, *p < 0.05.

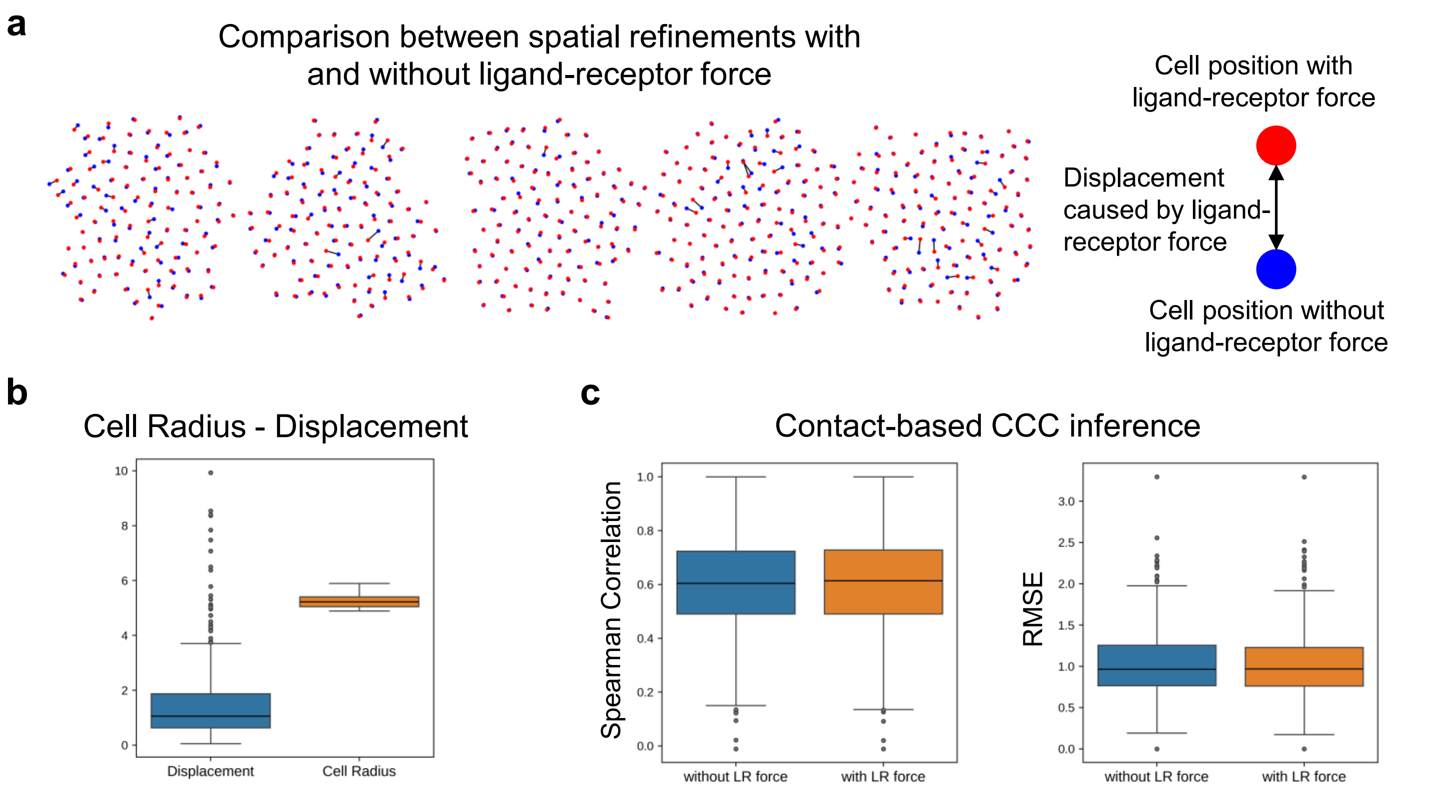

Supplementary Figure 8

Impact of ligand–receptor force on spatial refinement and Contact-based CCC inference

a, Spatial distributions of cells reconstructed by CellRefiner with or without ligand-receptor force. Blue dots represent cell positions obtained with the ligand-receptor force. Red dots represent cell positions obtained without the ligand-receptor force. Edges between dots indicate the displacement between the two simulations. **b**, Quantification of cell displacement compared with the cell radius. The displacement-to-cell-radius ratio below 1 indicates that ligand-receptor force induces minor perturbations in cell positions. **c**, Evaluation of predicted contact-based signaling on single-cell level using Spearman's rank correlation and root mean square error. Boxplot elements: center line, median; box limits, upper and lower quartiles; whiskers, 1.5x interquartile range; points, outliers. Source data are provided as a Source Data file.

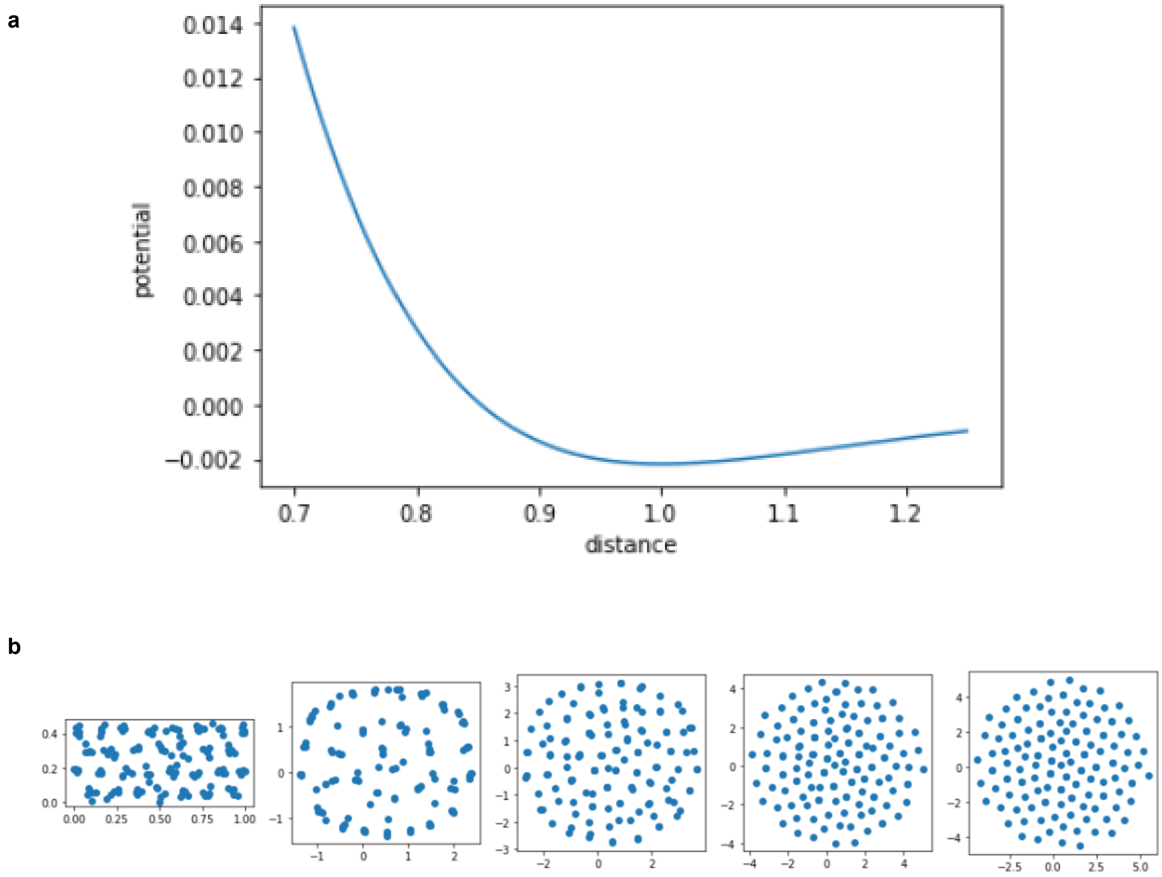

Supplementary Figure 9

Morse Potential Function

a, Morse potential function with potential well at 1. This is a dimensionless function scaled to the desired equilibrium distance between two particles, where the particles experience strong repulsion when closer than the equilibrium distance and mild attraction when further. **b,** Simulation of unconstrained particles under Morse potential (left to right). The particles eventually reach an equilibrium state where each particle is well-spaced from its neighbors.

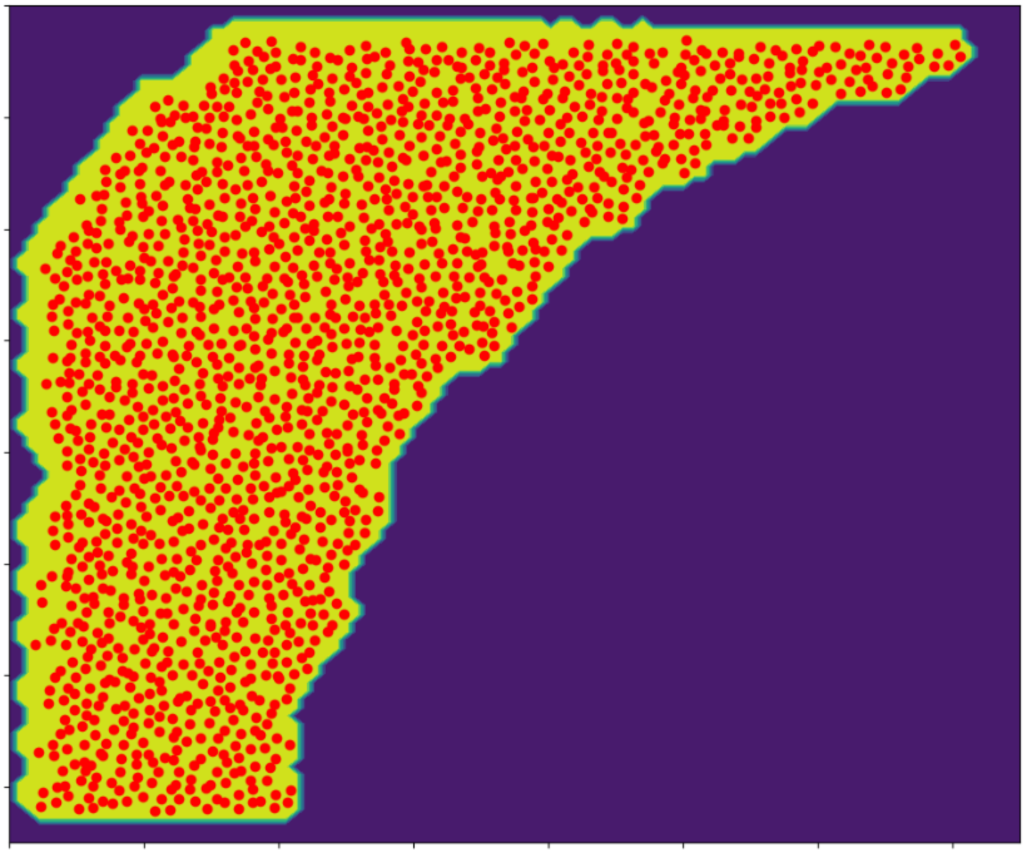

Supplementary Figure 10

Tissue Boundary

Tissue boundary restricting movement of CellRefiner cells (red circles) to the region of tissue (yellow) given by spatial data. This dataset is the mouse cortex Visium dataset paired with corresponding scRNA-seq.
