# Supplementary material for: Reconstructing single-cell resolution from spatial transcriptomics with CellRefiner
Source: Nat Commun. 2026 Feb 27;17:3304. doi: 10.1038/s41467-026-70090-2 (PMC13066420; doi:10.1038/s41467-026-70090-2)
Supplement: Supplementary file 2 — Reporting Summary [file 41467_2026_70090_MOESM2_ESM.pdf]

nature portfolio

Corresponding author(s): Qing Nie, Zixuan Cang

Last updated by author(s): 1/23/2026

Reporting Summary

Nature Portfolio wishes to improve the reproducibility of the work that we publish. This form provides structure for consistency and transparency in reporting. For further information on Nature Portfolio policies, see our Editorial Policies and the Editorial Policy Checklist.

Statistics

For all statistical analyses, confirm that the following items are present in the figure legend, table legend, main text, or Methods section.

n/a	Confirmed	
☐	☒	The exact sample size (n) for each experimental group/condition, given as a discrete number and unit of measurement
☒	☐	A statement on whether measurements were taken from distinct samples or whether the same sample was measured repeatedly
☒	☐	The statistical test(s) used AND whether they are one- or two-sided<br>*Only common tests should be described solely by name; describe more complex techniques in the Methods section.*
☒	☐	A description of all covariates tested
☒	☐	A description of any assumptions or corrections, such as tests of normality and adjustment for multiple comparisons
☐	☒	A full description of the statistical parameters including central tendency (e.g. means) or other basic estimates (e.g. regression coefficient) AND variation (e.g. standard deviation) or associated estimates of uncertainty (e.g. confidence intervals)
☒	☐	For null hypothesis testing, the test statistic (e.g. F, t, r) with confidence intervals, effect sizes, degrees of freedom and P value noted<br>*Give P values as exact values whenever suitable.*
☒	☐	For Bayesian analysis, information on the choice of priors and Markov chain Monte Carlo settings
☒	☐	For hierarchical and complex designs, identification of the appropriate level for tests and full reporting of outcomes
☐	☒	Estimates of effect sizes (e.g. Cohen's d, Pearson's r), indicating how they were calculated

Our web collection on statistics for biologists contains articles on many of the points above.

Software and code

Policy information about availability of computer code

Data collection	All data used are publicly available data.
Data analysis	The codes for the developed software and for reproducing the results are publicly available on Github at https://github.com/XiangyuKuang/cellrefiner, and on Zenodo at https://doi.org/10.5281/zenodo.18263150.

For manuscripts utilizing custom algorithms or software that are central to the research but not yet described in published literature, software must be made available to editors and reviewers. We strongly encourage code deposition in a community repository (e.g. GitHub). See the Nature Portfolio guidelines for submitting code & software for further information.

Data

Policy information about availability of data

All manuscripts must include a data availability statement. This statement should provide the following information, where applicable:

- Accession codes, unique identifiers, or web links for publicly available datasets
- A description of any restrictions on data availability
- For clinical datasets or third party data, please ensure that the statement adheres to our policy

The mouse hypothalamic preoptic region MERFISH original data is available at https://doi.org/10.5061/dryad.8t8s248 with preprocessed data available via the Squidpy package. The mouse visual cortex STARmap data is accessible via the STAGATE package. The mouse hippocampus Slide-seqV2 data is available at the Broad Institute Single Cell Portal (https://singlecell.broadinstitute.org/single_cell/study/SCP815/sensitive-spatial-genome-wide-expression-profiling-at-cellular-resolution#study-summary) with preprocessed data available via the Squidpy package. The human breast cancer Xenium data is available at the 10X Genomics

website (https://www.10xgenomics.com/products/xenium-in-situ/preview-dataset-human-breast). The mouse brain (sagittal posterior) Visium data is available at the 10X Genomics website (https://www.10xgenomics.com/resources/datasets/mouse-brain-serial-section-1-sagittal-anterior-1-standard-1-1-0). Corresponding scRNA-seq data is available at the Gene Expression Omnibus (GEO, https://www.ncbi.nlm.nih.gov/geo) under accession code GSE115746. Both 10X Visium and 10X Chromium samples for the lymph node are available at https://github.com/romain-lopez/DestVI-reproducibility. Human SCC Visium data and scRNA-seq data are available at GEO under accession code GSE144240. seqFISH+ mouse cortex data is available at https://github.com/CaiGroup/seqFISH-PLUS. The mouse kidney Visium data is available at the 10X Genomics website (https://www.10xgenomics.com/datasets/mouse-kidney-section-coronal-1-standard-1-1-0). The corresponding scRNA-seq data is available at GEO under accession code GSE117089. The ligand-receptor pairs with secreted ligands, as categorized in the CellChatDB, were used and can be accessed at http://www.cellchat.org/cellchatdb/. Source data are provided with this paper.

Research involving human participants, their data, or biological material

Policy information about studies with human participants or human data. See also policy information about sex, gender (identity/presentation), and sexual orientation and race, ethnicity and racism.

Reporting on sex and gender	Not applicable as this work only uses publicly available data.
Reporting on race, ethnicity, or other socially relevant groupings	Not applicable as this work only uses publicly available data.
Population characteristics	Not applicable as this work only uses publicly available data.
Recruitment	Not applicable as this work only uses publicly available data.
Ethics oversight	Not applicable as this work only uses publicly available data.

Note that full information on the approval of the study protocol must also be provided in the manuscript.

Field-specific reporting

Please select the one below that is the best fit for your research. If you are not sure, read the appropriate sections before making your selection.

☒ Life sciences ☐ Behavioural & social sciences ☐ Ecological, evolutionary & environmental sciences

For a reference copy of the document with all sections, see nature.com/documents/nr-reporting-summary-flat.pdf

Life sciences study design

All studies must disclose on these points even when the disclosure is negative.

Sample size	Not applicable as this work only uses publicly available data.
Data exclusions	No data was excluded.
Replication	The presented results can be reproduced using the corresponding Jupyter notebooks available at https://github.com/XiangyuKuang/cellrefiner
Randomization	Not applicable as this work only uses publicly available data.
Blinding	Not applicable as this work only uses publicly available data.

Reporting for specific materials, systems and methods

We require information from authors about some types of materials, experimental systems and methods used in many studies. Here, indicate whether each material, system or method listed is relevant to your study. If you are not sure if a list item applies to your research, read the appropriate section before selecting a response.

Materials & experimental systems

n/a	Involved in the study	
☒	☐	Antibodies
☒	☐	Eukaryotic cell lines
☒	☐	Palaeontology and archaeology
☒	☐	Animals and other organisms
☒	☐	Clinical data
☒	☐	Dual use research of concern
☒	☐	Plants

Methods

n/a	Involved in the study	
☒	☐	ChIP-seq
☒	☐	Flow cytometry
☒	☐	MRI-based neuroimaging

Plants

Seed stocks

Report on the source of all seed stocks or other plant material used. If applicable, state the seed stock centre and catalogue number. If plant specimens were collected from the field, describe the collection location, date and sampling procedures.

Novel plant genotypes

Describe the methods by which all novel plant genotypes were produced. This includes those generated by transgenic approaches, gene editing, chemical/radiation-based mutagenesis and hybridization. For transgenic lines, describe the transformation method, the number of independent lines analyzed and the generation upon which experiments were performed. For gene-edited lines, describe the editor used, the endogenous sequence targeted for editing, the targeting guide RNA sequence (if applicable) and how the editor was applied.

Authentication

Describe any authentication procedures for each seed stock used or novel genotype generated. Describe any experiments used to assess the effect of a mutation and, where applicable, how potential secondary effects (e.g. second site T-DNA insertions, mosiacism, off-target gene editing) were examined.
